# Monitoring nutrients in plants with genetically encoded sensors: achievements and perspectives

**DOI:** 10.1093/plphys/kiad337

**Published:** 2023-06-12

**Authors:** Mayuri Sadoine, Roberto De Michele, Milan Župunski, Guido Grossmann, Vanessa Castro-Rodríguez

**Affiliations:** Institute of Cell and Interaction Biology, Heinrich-Heine Universität Düsseldorf, Düsseldorf 40225, Germany; Institute of Biosciences and Bioresources, National Research Council of Italy, Palermo 90129, Italy; Institute of Cell and Interaction Biology, Heinrich-Heine Universität Düsseldorf, Düsseldorf 40225, Germany; Institute of Cell and Interaction Biology, Heinrich-Heine Universität Düsseldorf, Düsseldorf 40225, Germany; Cluster of Excellence on Plant Sciences, Heinrich-Heine Universität Düsseldorf, Düsseldorf 40225, Germany; Departamento de Biología Molecular y Bioquímica, Facultad de Ciencias, Universidad de Málaga, Málaga 29071, Spain

## Abstract

Understanding mechanisms of nutrient allocation in organisms requires precise knowledge of the spatiotemporal dynamics of small molecules in vivo. Genetically encoded sensors are powerful tools for studying nutrient distribution and dynamics, as they enable minimally invasive monitoring of nutrient steady-state levels in situ. Numerous types of genetically encoded sensors for nutrients have been designed and applied in mammalian cells and fungi. However, to date, their application for visualizing changing nutrient levels in planta remains limited. Systematic sensor-based approaches could provide the quantitative, kinetic information on tissue-specific, cellular, and subcellular distributions and dynamics of nutrients in situ that is needed for the development of theoretical nutrient flux models that form the basis for future crop engineering. Here, we review various approaches that can be used to measure nutrients in planta with an overview over conventional techniques, as well as genetically encoded sensors currently available for nutrient monitoring, and discuss their strengths and limitations. We provide a list of currently available sensors and summarize approaches for their application at the level of cellular compartments and organelles. When used in combination with bioassays on intact organisms and precise, yet destructive analytical methods, the spatiotemporal resolution of sensors offers the prospect of a holistic understanding of nutrient flux in plants.

## Introduction

**Figure 1.**
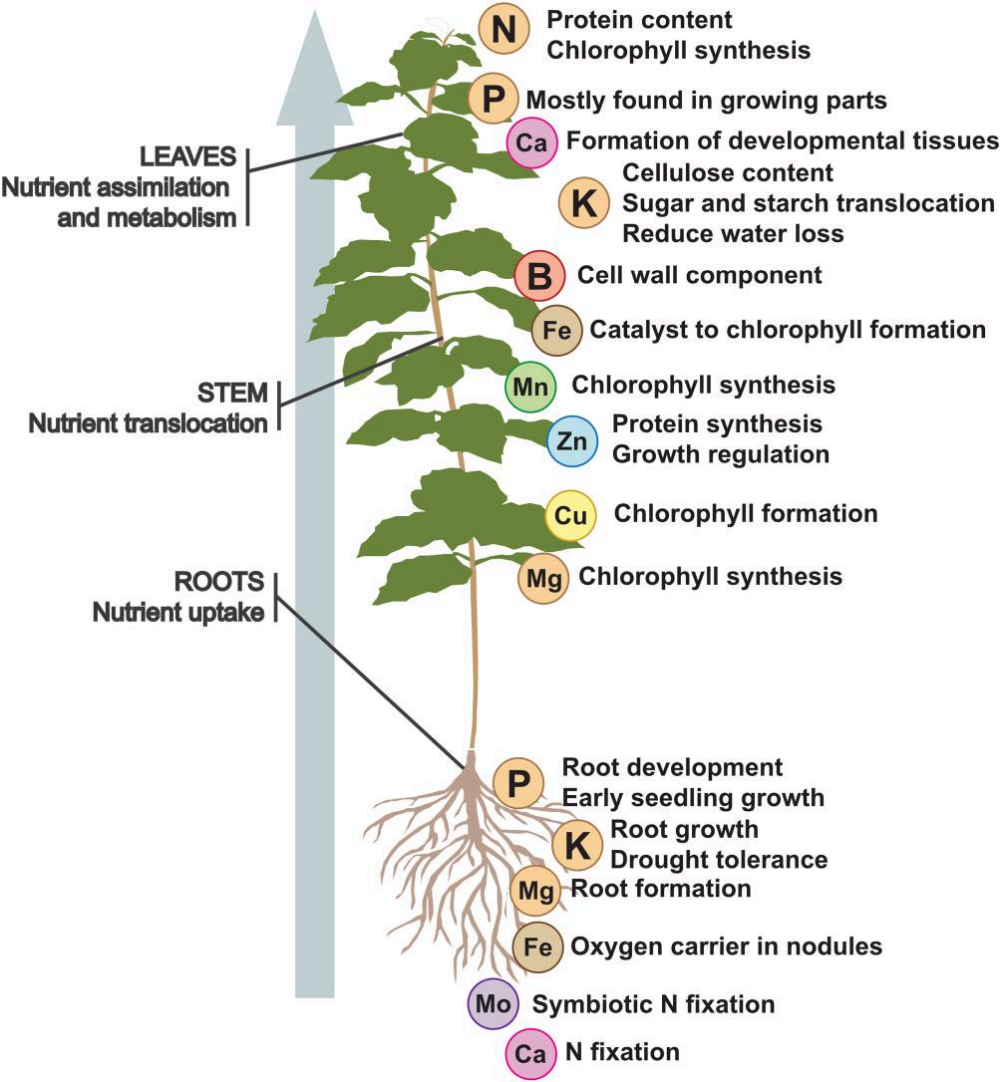
Nutrient distribution in plants. Schematic representation of nutrient distribution within a plant. Macro- and micronutrients are involved in multiple processes that occur in different plant areas. For example, P, K, Fe, and Ca are involved in the aerial and ground parts and act as key factors during such processes as growth and development, sugar and protein translocation, chlorophyll formation, and N fixation, as well as cellulose content. N, B, Mn, Zn, and Cu are mostly found in growing parts and are important to chlorophyll, protein, and sugar synthesis, as well as plant growth regulation. Mg and Mo are both primarily found in the roots, where they are involved in root formation and symbiotic N fixation. Additionally, Mg is required to synthesize chlorophyll in photosynthetic tissues.

As sessile organisms, plants depend on nutrient availability in the surrounding soil^1^. Mineral nutrients are mostly absorbed from soil via plant roots and distributed through vascular transport (Table 1 and Fig. 1). Efficiency of nutrient acquisition is affected by soil composition and root structural adaptations to nutrient limitation (Shahzad and Amtmann 2017). Plasticity of the root system architecture (RSA) allows plants to access essential nutrients whose deficiency affects development, growth, and fertility (López-Bucio et al. 2003). Plants show different responses to specific nutrient deficiencies, which vary between species. Within cells, each organelle performs specific physiological roles. Knowing the nutrient concentrations in each compartment helps to determine which process the organelle participates in, and the cell metabolic stage.

**Advances**

- Over the past 3 decades, many sensors were successfully engineered for monitoring nutrients and metabolites in living cells. Some of the advances made in the different biology fields have proven their utility and potential when used with rational to address key biological questions.
- Sensors are unique tools for monitoring analytes in real time in living cells without interfering with the organism physiology making them particularly useful for investigating questions related to growth and development.
- Rational/semirational design and random mutagenesis are important approaches used in sensor development for rendering them relevant under physiological conditions.
- Advanced technologies such as AI-based approaches and advanced imaging systems will be important for dealing with obstacles to a more systematic implementation of sensors in plants including crops.

**Table 1.** Macronutrient concentrations (mM) in plant cell compartments

		Cyt	Vac	Mit	Plast	Organ/plant	Cell type/extract	Techniques	Ref.
N	NO_3_^−^	1 to 6	5 to 75	ND	5	Tobacco plants, barley, maize, pea, soybean, rice, spinach plants/barley roots/leaf under light-dark conditions *Arabidopsis*/living *Arabidopsis* root cells/Arabidopsis guard cells	Plant extracts barley root cortical and epidermal cells/epidermal/mesophyll/Arabidopsis guard cells	NRA-based/microelectrodes/14N-NMR/sensor	(Dechorgnat et al. 2011; Demes et al. 2020; Chen et al. 2022)
	NH_4_^+^	0.005 to 1.5	15 to 19	5 to 10	ND	Corn shoots/maize and rice roots	Shoot extracts/roots extracts	Enzyme assay/NMR/radioisotope	(Yamaya et al. 1984; Miller et al. 2001)
Pi		0.1 to 12	0.01 to 13.56	4 to 7.2 (Mit + Plast)	4 to 7.2 (Mit + Plast)	*Acer pseudoplatanus* and *Arabidopsis* cells/living *Arabidopsis* root cells/soybean leaves	Sycamore and *Arabidopsis* cells/root cells/soybean leaves cells	Colorimetric assay/^31^P-NMR/mass spectrometry/radioisotopes/sensors	(Lauer et al. 1989; Pratt et al. 2009; Mukherjee et al. 2015; Sahu et al. 2020)
K^+^		100 to 200	10 to 500	ND	100 to 200	*Arabidopsis* root hair/*Arabidopsis* roots	*Arabidopsis* roots	Chemical dye/sensors	(Hawkesford et al. 2012; Sun et al. 2020; Wang et al. 2021)
Na^+^		1 to 10	ND	ND	ND	Cell suspensions of tobacco plants/barley root apex/*Arabidopsis* root cells/*Arabidopsis* leaf, root hair, vascular bundle, and root tips/wheat roots	Tobacco plant cells/*Arabidopsis* epidermal root cells/wheat root cells	X-ray/microelectrodes/chemical dye	(Binzel et al. 1988; Park et al. 2009; Wu et al. 2018)
Mg^2+^		0.2 to 0.4	5 to 80	0.2 to 0.5	10 to 15	Root tips/epidermal root cells/mycorrhizal roots/epidermis leaves/beet roots/spruce needles/spinach chloroplast	Mung bean/*Arabidopsis*/Norway spruce/barley/red beet/mesophyll, endodermis, parenchyma cells/intact chloroplast	^31^P-NMR/chemical dye/isotopic tracer/microelectrodes/X-ray microanalyses (XRMA)/pH electrode measurement	(Hermans et al. 2013)
Ca^2+*^		1 × 10^−4^	0.2 to 50	1 × 10^−4^ to 6 × 10^−4^	5 × 10^−4^	*Arabidopsis* leaves/*Arabidopsis* leaves/roots/soybean/tobacco/*Pisum sativum*	Mesophyll cell/*Arabidopsis* leaves/roots/cell suspension soybean/protoplast of tobacco/etiolated pea stems	XRMA/sensors	(Costa et al. 2018)
S		1 to 11	6 to 75	ND	4 to 12	Barley leaves, oat roots, roots and leaves of *Macroptilium atropurpureum*	Mesophyll and epidermal vacuoles in leaves, microsomal vesicles of oat roots	Isotopic tracer	(Churchill and Sze 1984; Buchner et al. 2004)
Cl^−^		10	50 to 150	ND	ND	*Sinapis alba*, rootstock of *Cleopatra mandarin*, *Rangpur lime*, *Rough lemon*, and *Carrizo citrange*, shoots of plantlets of *Arabidopsis*, leaf cell of tobacco, mature and young leaves, stem, and roots of tomato plants; *Arabidopsis* roots	Root hair cells, total tissue, shoots of *Arabidopsis*, shoots and roots of tobacco, and roots of *Arabidopsis*	Microelectrodes, colorimetric assay, and sensors	(Felle 1994; Broadley et al. 2012; Colmenero-Flores et al. 2019)

Nonexhaustive list of nutrient concentrations (mM) in different plant cell compartments.

Cyt: cytoplasm; Vac: vacuole; Mit: mitochondrion; Plast: plastidic; ND: not determined. *Range/estimation of resting free Ca^2+^

**Figure 2.**
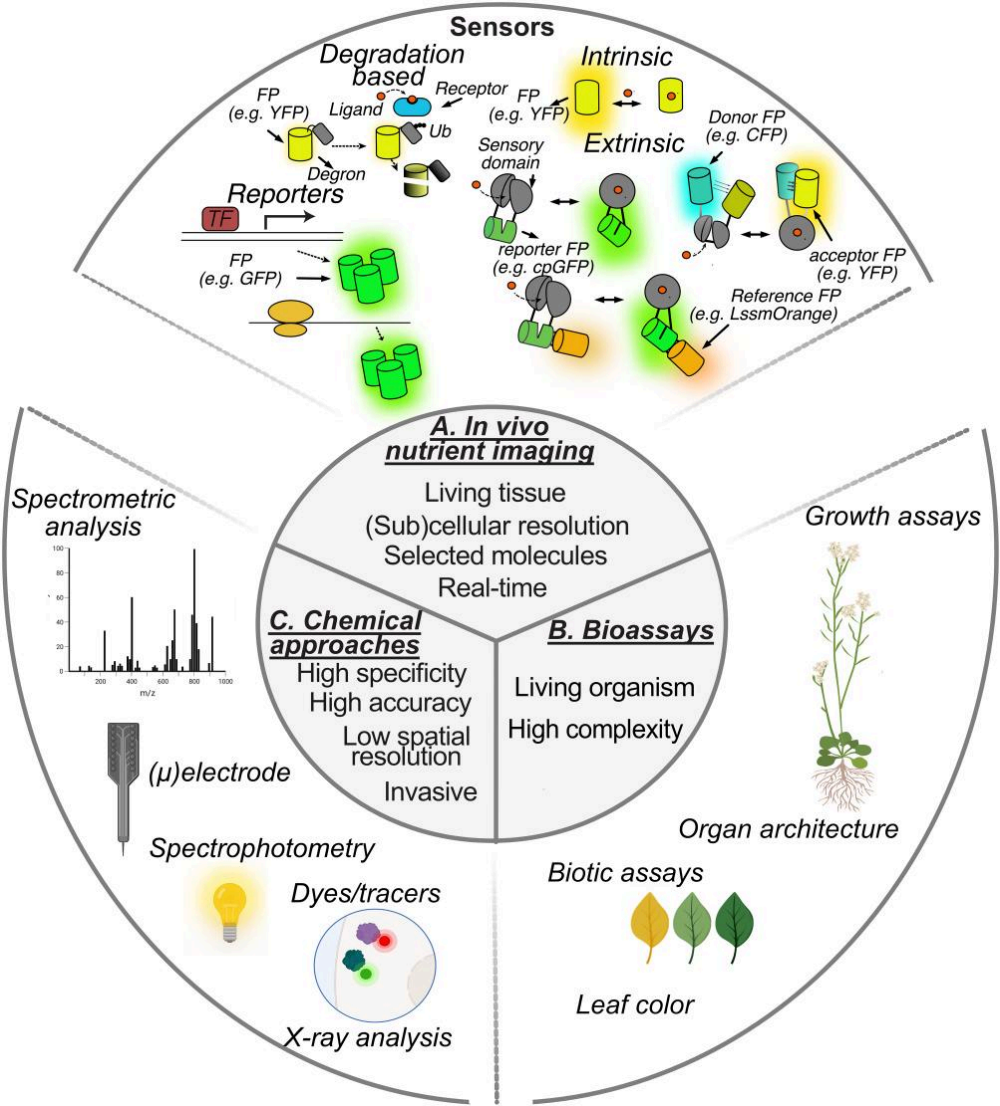
Holistic nutrient analysis approaches in plants. **A)** In vivo nutrient imaging including sensor imaging with indirect sensors, i.e. transcriptional fluorescent reporters, translational fluorescent reporters, Degron-FP fusion, and direct sensors, i.e. intrinsic sensors using FP as recognition element, extrinsic sensors including FRET-based sensors and intensiometric cpFP-based sensor; and ratiometric Matryoshka design with nested reference FP. **B)** Bioassays, i.e. organ architecture, leaf color, growth assays, and biotic assays. **C)** Chemical approaches, i.e. spectrometric analysis (e.g. ICP-OES, ICP-MS, and AAS), ()electrode, spectrophotometry, dye/tracer (e.g. radiotracer and fluorescent/chemical dyes), and X-ray analysis (e.g. XRMA). ICP-OES, Inductively Coupled Plasma Optical Emission Spectrometry; ICP-MS, inductively coupled plasma mass spectrometry; AAS, atomic absorption spectrometry; XRMA, X-ray microanalysis; TF, transcription factor.

**Figure 3.**
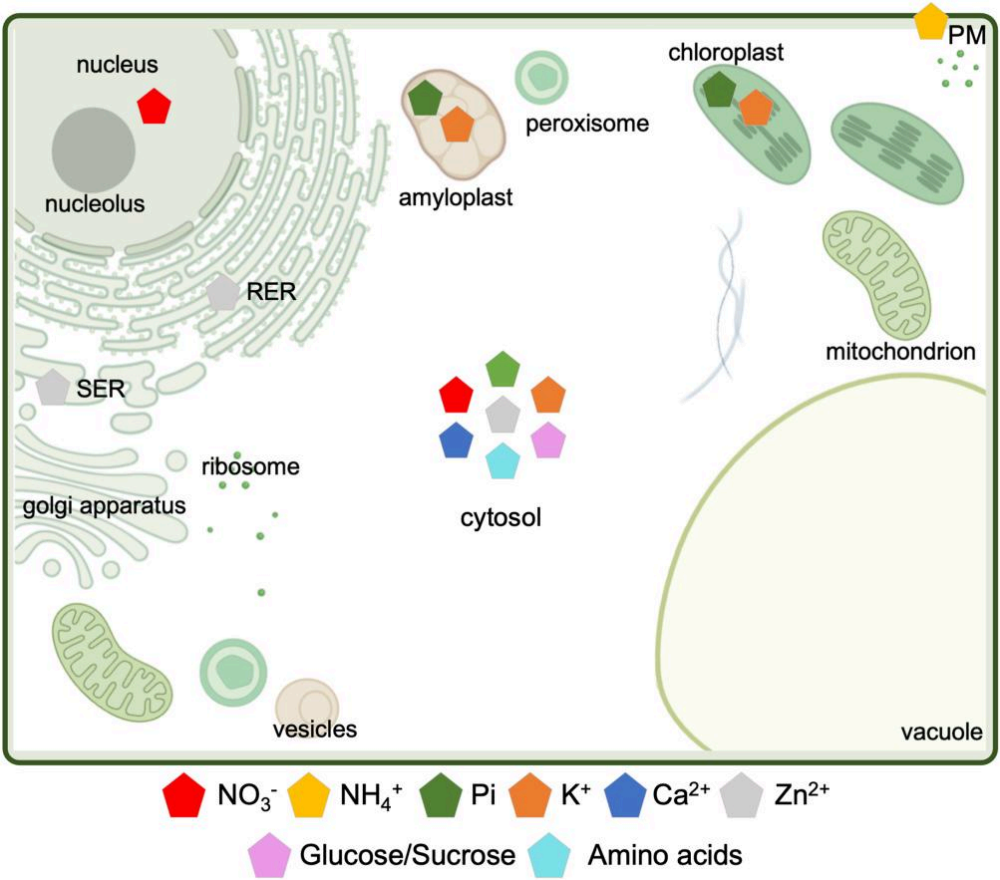
Subcellular targeting of sensors in plants. Subcellular compartmentalization of nutrients that have been investigated using targeting of sensors to specific organelles in plant cells. RER, rough endoplasmic reticulum; SER, smooth endoplasmic reticulum. Distribution of sensors targeting the plastids (amyloplast and chloroplast), plasma membrane PM, or no target (cytosol) are represented.

Quantitative measurement of nutrient concentrations has been typically performed by nutrient-level determination based on chemical-based approaches including enzymatic assays (Roskoski 2007), mass spectrometry (Jorge et al. 2016), high-performance liquid chromatography (Linskens and Jackson 2012), radioactive tracer (Martin and Russell 1950), or ion-selective microelectrodes (Miller and Smith 2012, p.). These methods have high accuracy and sensitivity, but they lack (sub)cellular and temporal resolution in vivo (Fig. 2). The development of genetically encoded fluorescent sensors has opened new routes to tracing nutrient and metabolite dynamics (Table 2 and Fig. 2). Initially employed for cytosolic measurements, sensors can be targeted to subcellular compartments, allowing monitoring of molecules in space and time (Bermejo et al. 2011) (Table 2 and Fig. 3). First-generation sensors were based on Förster resonance energy transfer (FRET) between fluorescent proteins (FPs) fused to binding proteins (BPs) undergoing conformational changes upon ligand binding (Allen et al. 1999b) (Box 1). Later, scientists engineered conformationally sensitive, circularly permutated green FP (cpGFP) allowing the design of intensiometric sensors with improved signal-to-noise ratio (Baird et al. 1999). The readout of single-FP sensors is sensitive to changes in expression levels, but some are intrinsically ratiometric due to dual excitation or emission spectra (Ast et al. 2015; Li et al. 2021); others can harbor a second reference FP (Ast et al. 2017; Waadt et al. 2017; Li et al. 2021) fused to the sensor temini or nested within the sensor (Ast et al. 2017) (Box 1).

Box 1. **Outstanding questions**

- Despite the fact that a long list of sensors is available, they have not yet lead to substantial advances in many aspects of plant science likely due to a lack of relevance of existing sensors and/or challenges in their implementation in plants. How can we facilitate the use of sensors in plants?
- Can sensor development and utilization be accelerated by using advanced emerging technologies such as AI-based approaches?
- What are the genetic and environmental factors that influence the expression and performance of genetically encoded nutrient sensors in plants, and how a better understanding of these factors can lead to more accurate and reliable nutrient monitoring in crops?
- How can we develop standardized procedures and integrate biosensor approaches into broader analytical pipelines to obtain reproducible and quantitative information on steady-state levels of nutrients in crops?

FPs fused to proteins or promoters (i.e. translational and transcriptional reporters) are frequently used for visualizing localization and expression of proteins. FPs have also been used in the design of genetically encoded fluorescent-based sensors allowing scientists to monitor analyte steady-state concentration changes in cells. Sensors are commonly used to convert molecular events such as protease activity, protein–protein interactions, and conformational changes into optical signals (Frommer et al. 2009). The optical readout can be either ratiometric or intensiometric. Intensiometric sensors rely on measuring changes in fluorescence intensities of a single FP rendering them, unfortunately, sensitive to variations in sensor abundance (e.g. in differential gene expression). This crosstalk can be corrected by using ratiometric sensors, with the simultaneous measurement of signal fluorescence intensities of at least 2 spectrally separated FPs, with the emission intensity ratios representing the degree of substrate bound to the sensor. However, using ratiometric sensors limits the coexpression of multiple sensors as well as the use of other FP fusions or dyes. One large class of ratiometric sensors are FRET-based sensors. The main aspect of FRET-based sensors is their capability to respond with ratiometric fluorescence intensity changes due to changes in FRET efficiency (Frommer et al. 2009). The second major class of sensors is made by single cpFP-based sensors that rely on the sensitivity of a cpFP to translate conformational changes of a sensory domain into fluorescence intensity changes. One advantage of cpFP-based sensors lies in the fact that they often display larger signal-to-noise changes in their response (Frommer et al. 2009). However, they are also prone to abundance-related artefacts, as mentioned above. Advanced ratiometric designs make use of a reference FP, such as a C-terminal mTurquoise (Waadt et al. 2017) or a nested LSSmOrange in the Matryoshka concept (Ast et al. 2017). One final main advantage of using genetically encoded sensors is the ability to target them at cell compartments. This renders achievable to understand nutrient status at subcellular levels. Nuclear targeting is possible by fusing to a signal peptide (e.g. NLS) and improves substantially the visualization of cytosolic sensors often impaired by large vacuoles (Rizza et al. 2021). Using a similar approach, sensors have been targeted to various organelles and microdomains or even fused to other proteins such as transporters (Tay et al. 2012). One potential issue is that sensor expression might affect trafficking or may show a sponge effect depending on the localization (Castro-Rodríguez et al. 2022).

**Table 2.** Overview of genetically encoded nutrient sensors and their use in previous studies (Sadoine et al. 2021a)

Nutrient		Ligand	Sensor	Affinity (mM)	Technology	Organism	Location	Ref.
N	Nitrate	NO_3_^−^	sNOOOpy (NO_3_^−^; NO_2_^−^)	0.0395; 0.256	FRET	Bacteria/mammals	Cyt	(Hidaka et al. 2016)
			ClopHensor	5.3	Single FP	Mammals/plants	Cyt	(Demes et al. 2020)
			FLIP-NT	1 × 10^−3^/0.056	FRET	Bacterial/yeasts	Cyt	(Fatima et al. 2020)
			NitraMeter3.0/nlsNitraMeter3.0	0.09/0.13	FRET	Plants	Cyt, Nuc	(Chen et al. 2022)
			NiTracs	0.075/3.8	FRET	Yeasts/oocytes	Mem	(Ho and Frommer 2014; Chen and Ho 2022)
	Ammonium	NH_4_^+^	AmTracs, MepTrac	0.055/0.1	Single FP	Yeasts/oocytes/plants	Mem	(De Michele et al. 2013)
			deAmTracs	0.036/0.054	Single FP (ratiometric)	Yeasts	Mem	(Ast et al. 2015)
			AmTryoshkas	0.05	Dual FP	Yeasts/oocytes	Mem	(Ast et al. 2017)
Inorganic phosphate (Pi)		Pi	Pi-FRET (FLIPPi)	7.7 × 10^−4^, 4 × 10^−3^, 5 × 10^−3^, 0.2, 6, 30	FRET	Mammals/plants	Cyt	(Gu et al. 2006; Mukherjee et al. 2015; Assunção et al. 2020)
			cpFLIPPi	0.08, 0.2, 1.6, 3.5, 4.8, 5.3, 6.4, 11.0	Single FP	Plants	Cyt/Plast	(Mukherjee et al. 2015; Sahu et al. 2020)
Potassium		K^+^	GEPIIs	3.24, 4.39, 8.59, 27.43	FRET	Bacteria/mammals	Cyt/Nuc/Mit	(Bischof et al. 2017)
					FRET	Plants	Cyt	(Wang et al. 2021)
			KRaION1	39 to 112	FRET	Mammals	Cyt	(Torres Cabán et al. 2022)
			KIRIN1	1.66	FRET	Mammals	Cyt	(Shen et al. 2019)
			KIRIN1-GR	2.56	FRET	Mammals	Cyt	(Shen et al. 2019)
			GINKO1	0.42	Single FP (ratiometric)	Mammals	Cyt	(Shen et al. 2019)
			GINKO2	15.3	Single FP (ratiometric)	Bacteria/plants/mammals	Cyt	(Wu et al. 2022b)
Magnesium		Mg^2+^	MagFRET-1	1.5 × 10^−4^	FRET	Mammals	Cyt/Nuc	(Lindenburg et al. 2013)
			MagIC	2	Dual FP	Mammals	Cyt/Nuc Mit/ER	(Koldenkova et al. 2015)
Calcium		Ca^2+^	Aequorin	ND	Single FP	Plants	Cyt	(Knight et al. 1991)
			GCaMPs	2 × 10^−5^, 3 × 10^−4^	Single FP	Mammals/bacteria	Cyt	(Akerboom et al. 2009, 2012; Cho et al. 2017)
			GECOs	1 × 10^−5^ to 1 × 10^−4^	Single FP	Mammals/worms/plants	Cyt	(Waadt et al. 2017)
			CatchER	0.18	Single FP	Mammals	ER/SR	(Tang et al. 2011, p. 2)
			Cameleons	1 × 10^−8^, 0.042, 1	FRET	Mammals	Cyt/Nuc/ER	(Miyawaki et al. 1997)
			FRET TN-XXL	8 × 10^−4^	FRET	Bacteria/mammals	Cyt	(Thestrup et al. 2014)
			Twitch	1.5 × 10^−6^ to 2.57 × 10^−3^	FRET	Mammals	Cyt	(Thestrup et al. 2014)
			CaMPARI	7.4 × 10^−6^ to 1.3 × 10^−7^	Dual FP	Mammals	Cyt	(Fosque et al. 2015; Ebner et al. 2019)
			MatryoshCaMP6s	2 × 10^−4^	Dual FP	Bacteria/mammals/plants	Cyt	(Ast et al. 2017)
			NEMO	1.41 × 10^−4^ to 5.57 × 10^−4^	Single FP	Mammals	Cyt	(Li et al. 2022)
Chloride		Cl^−^	Clomeleon	160	FRET	Bacteria/mammals/plants	Cyt	(Bregestovski and Arosio 2011)
			ClopHensor	50	Single FP	Mammals/plants	Cyt	(Arosio et al. 2010)
			Cl-sensor	30	Single FP	Mammals	LDCV	(Bregestovski and Arosio 2011)
			ChlorON	10	Single FP	Mammals	Cyt	(Tutol et al. 2022)
			E^2^GFP	17.5 to 163	Single FP	Plants	Cyt	(Waadt et al. 2020)
Manganese		Mn	PET sensors, iLovU	ND	Single FP	Bacteria	Cyt	(Liu et al. 2014)
Zinc		Zn^2+^	ZapCY1/ZapCY2	2.5 × 10^−9^ to 8 × 10^−8^	FRET	Mammals	Cyt, ER	(Qin et al. 2011)
			eCALWYs	2 × 10^−9^, 9 × 10^−9^, 4 × 10^−8^, 6 × 10^−7^, 1.85 × 10^−6^, 3 × 10^−6^	FRET, BRET	Mammals, Plants	Cyt, ER	(Vinkenborg et al. 2009; Lanquar et al. 2014; Aper et al. 2016)
			eZinChs	1 × 10^−6^, 2.3 × 10^−6^	FRET, BRET	Mammals	Cyt, ER	(Evers et al. 2007; Aper et al. 2016)
			Zn72R	0.056	FRET	Mammals	Cyt	(Xian et al. 2020)
			GZnPs	6 × 10^−8^, 3.52 × 10^−7^, 1 × 10^−6^	Single FP, Dual FP	Mammals	Cyt, Mem, Mito (Matrix and IMS)	(Qin et al. 2016; Fudge et al. 2018)
			ZnGreens, ZnRed	6 × 10^−4^, 0.02, 1.66 × 10^−4^	Single FP	Mammals	Cyt, Cell Surface, Nuc	(Chen and Ai 2016)
			ZIBG1/ZIBG2	2.81 × 10^−3^/2.8 × 10^−4^	Single FP	Mammals	Cell Surface	
Copper		Cu	CusSR	ND	Dual FP	Bacteria	Cyt	(Ravikumar et al. 2012)
			AMT1-FRET	2 × 10^−15^	FRET	Bacteria/mammals	Cyt	(Wegner et al. 2010)
			Ace1-FRET Mac1-FRET Cup1-FRET, Crs5-FRET	4.7 × 10^−18^, 9.7 × 10^−20^, 3.6 × 10^−18^, 2.3 × 10^−18^	FRET	Yeasts	Cyt	(Wegner et al. 2011)
Molybdenum		Mo	MolyProbe	4.7 × 10^−5^	FRET	Plants/yeasts	Cyt	(Nakanishi et al. 2013; Oliphant et al. 2022)

Nonexhaustive list of nutrient sensors; affinity ND: no determinate. Mem: membrane; Cyt: cytosol; Plast: plastid; Nuc: nucleus; Mit: mitochondrion; LDCV: large dense-core vesicle; ER: endoplasmic reticulum.

The work on the development of calcium sensors and later bacterial periplasmic BPs (PBPs) has laid the basis for sensor imaging of nutrients and metabolites in living cells (Lager et al. 2006; Sadoine et al. 2021b). Sensors have been used widely across organisms as exemplified by glucose sensors (Bermejo et al. 2013). Several studies support their ability to monitor analyte levels and identify regulatory/metabolic networks, transport processes, signaling, and cell-to-cell communication (Chen et al. 2010; Toyota et al. 2018).

## Nitrogen (N)

As component of proteins, nucleic acids, and secondary metabolites, N is 1 main component of plants. N plays roles in protein synthesis, energy production, and metabolism. N is transported by membrane transporters in inorganic or organic forms and then assimilated (Reid and Hayes 2003). Plants have N levels of 1% to 5% of dry weight. For several plants, when N concentration decreases, deficiency symptoms appear affecting yield, growth, and development (de Bang et al. 2021). Although N_2_ comprises 78% of the atmosphere, it is usually limiting for plants. Indeed, molecular dinitrogen is not directly available to plants and needs to be reduced by N-fixing bacteria, free living, or in symbiotic association with plant roots. Plants can uptake N in the form of ammonia, nitrate, urea, or organic compounds. Since N sources are usually limited in soil and artificial N fixation for fertilizer production is an industrial process with high economic and environmental impacts, a goal for plant breeders/scientists is to increase the N use efficiency (NUE) of crops. Traditional techniques for measuring concentrations of N forms in plants include colorimetric, chromatographic, fluorimetric, and radiotracer assays. For ammonia, the widely used methods based on colorimetric Berthelot reaction and ion chromatography suffer from interference caused by amino acids, amines, amides, and proteins (Schjoerring et al. 2002). Fluorimetric assays by derivatization of ammonium with o-phthalaldehyde (OPA) with 2-mercaptoethanol as a reductant are more selective and sensitive and can be applied to small sample volumes (Schjoerring et al. 2002). Likewise, for nitrate quantification, the most reliable techniques are ion chromatography and in vitro enzymatic reduction by nitrate reductase (Cruz and Martins Loução 2002). However, all these techniques are destructive, have lowly sensitive, and are prone to artefacts if sample material is not stabilized, since amino acids and other labile N metabolites can degrade, affecting N pools.

Although sensors have potential to aid the development of strategies for NUE optimization and screening tests, the current toolbox for inorganic N compounds is limited (Table 2). No sensor is available yet for soluble ammonium or urea. Conversely, both nitrate and nitrite are targets of the fluorescent sensor sNOOOpy, based on the bacterial 2-component transcriptional system involved in nitrate assimilation in *Bradyrhizobium japonicum* (Hidaka et al. 2016). In its design, NasT and NasS, partners of the 2-component system, are fused to the FRET pair CFP/cpVenus. Binding of nitrate/nitrite promotes the dissociation of the complex, and decrease in FRET between the FPs. The in vitro *K_d_* of sNOOOpy for nitrate and nitrite are 39.5 and 256 *µ*M, respectively, with a dynamic range of 1 *µ*M–1 mM for nitrate, while for nitrite, it is 1 order of magnitude higher. sNOOOpy has not yet been used in plants, but its affinity might prove inadequate, since estimated nitrate cytosolic concentrations exceed 1 mM (Table 1). To be used in plants, it would be useful to create variants with reduced affinity using semirational or random mutagenesis that have been successful for other sensors (Lindenburg and Merkx 2014). Another potential limitation of sNOOOpy for in vivo studies is its bicomponent structure. For use in mammalian cells, the 2 components were expressed as a single polypeptide that was later cleaved by endogenous peptidases (Hidaka et al. 2016). However, degradation might affect differently the 2 components, generating artifacts. Another way to improve sNOOOpy would be to fuse the moieties in a single polypeptide chain. Finally, another drawback of sNOOOpy for plant studies is its inability to discriminate between nitrate and nitrite, since they are usually both present in plant cells and subject to different regulation.

Recently, it was found that ClopHensor, a sensor originally tested in mammalian cells to monitor both Cl^−^ and pH, is actually sensitive to nitrate (Arosio et al. 2010). With a *K_d_* for nitrate of 5.3 mM and a dynamic range of 0.6 to 48 mM, ClopHensor is suitable for plants. Expression of ClopHensor in Arabidopsis guard cells allowed monitoring fluctuations in nitrate and pH in relation to the activity of the Cl channel *At*CLCa (Demes et al. 2020). While it may be interesting to measure more parameters with 1 sensor, it would be ideal to have a sensor specific to each nutrient. Another family of nitrate sensors was recently developed, based on the PBP NrtA fused between the FRET pair CFP/YFP. The FLIP-NT *K_d_* is 1 to 56 *µ*M, with a dynamic range of 0.2 to 80 *µ*M (Fatima et al. 2020). The high affinity and short dynamic range of FLIP-NTs, however, make them unlikely to be useful in plants, since the estimated millimolar cytosolic concentration of nitrate would saturate the sensor (Table 2). Moreover, potential cross-reactions of FLIP-NT against nitrite, and other ions, have not been tested (Fatima et al. 2020). Latest advances in the development of nitrate sensors relied on NitraMeter3.0 (Chen et al. 2022), developed by fusing the FRET pair Aphrodite/Cerulean to the bacterial soluble receptor for nitrate/nitrite NasR. NiTraMeter3.0 shows affinities of 90 *µ*M for nitrate and 2 *µ*M for nitrite in vitro, but when expressed in Arabidopsis roots, it responded to exogenous nitrate pulses and not to nitrite addition. However, this could result from impaired nitrite import within cells. Therefore, caution is needed when interpreting measurements in cells where nitrite is present and might saturate the sensor. Surprisingly, NitraMeter3.0 was able to detect changes in nitrate levels, despite its relatively high affinity and the estimated millimolar cytosolic concentrations (Chen et al. 2022) (Table 1), suggesting that in vivo expression probably decreases its affinity more than what had been estimated (i.e. *K_d_* = 130 *µ*M).

Despite a paucity of sensors for soluble N nutrient, there are sensors for monitoring N uptake. The first of these so-called transport activity sensors is based on the ammonium transporter AMT1;3 from Arabidopsis, dubbed AmTrac (De Michele et al. 2013). By inserting a modified cpGFP in a central cytosolic loop of AMT1;3, it is possible to monitor ammonium transport by measuring fluorescence intensity. Sensor specificity, correlation between transporter affinities, and fluorescence responses, the behavior of inactive mutants and their suppressors all point to a model in which the movement of the transmembrane domains during ammonium transport is reflected in the opening of the chromophore niche in the cpGFP, affecting fluorescence. AmTrac variants based on other Arabidopsis and yeast AMT members (AmTrac1;2 and MepTrac) were created, using a similar strategy (De Michele et al. 2013). A limitation of AmTracs is the intensiometric response prone to artifacts due to sensor expression levels. Two strategies were adopted to render them ratiometric. First, it was observed that a series of AmTrac variants in the linkers between cpGFP and AMT moieties show 2 emission peaks, whose relative intensities change during ammonium transport. These sensors are named deAmTrac for their dual emission behavior (Ast et al. 2015). The second strategy, called Matryoshka, consisted in nesting a reference FP, LSSmOrange, into cpGFP, generating AmTryoshka sensors (Ast et al. 2017). Activity transporter sensors are a new paradigm for studying transport and might prove instrumental to identifying new regulation mechanisms. Notably, inhibition of the AmTryoshka1;3 fluorescence response in yeast by coexpressing calcineurin-B–like (CBL)-interacting protein kinase 15 (CIPK15) led to the identification of the kinase responsible for allosteric trans-inhibition of plant AMT activity triggered by ammonium supply (Chen et al. 2020), which is aimed at avoiding toxicity. NiTrac activity transporter sensors were later created for the Arabidopsis nitrate transporter NPF6.3 (Ho and Frommer 2014). NiTrac uses the classic sandwich design of 2 FRET FPs separated by NRT1.1/CHL1. Interestingly, NiTrac maintained the same double low-/high-affinity behavior of the parent transporter, making it suitable to study NRT1.1/CHL1 regulation. Recently, another Arabidopsis nitrate transporter, NPF1.3, was converted into a sensor using the same strategy (Chen and Ho 2022). So far, NiTracs have been characterized only in yeast and oocytes, and it is not known whether expression in plants affects the nitrate transport capacity of cells.

## Phosphorous (P)

P in the form of phosphate is a major component of biomolecules like nucleotides, phospholipids, and phosphoproteins. Deficiencies in Pi result in impaired growth, late flowering, and browning/wrinkling of leaves (de Bang et al. 2021). Plants can only assimilate P in its inorganic phosphate form (Pi), which is usually present in low concentrations (1 to 8 *µ*M), and rather immobile in soil, resulting in P deficiency (Smith et al. 2003). Unlike N, Pi is a finite resource, whose reservoirs in bedrock and guano deposits are rapidly depleted for the production of fertilizers (Cordell et al. 2009). This highlight the needs to optimize Pi usage and reduce nutrients lost via leaching. One of the most ancient strategy to cope with Pi limitation in soil is the establishment of arbuscular mycorrhizae (AM), a symbiosis occurring in 70% to 80% of plant species (Gianinazzi et al. 2010). Sensors for Pi, when expressed in plant roots, are excellent tools to study both direct and AM-mediated Pi uptake. Notably, Arabidopsis does not form AM associations, limiting its use for studies on plant–fungus symbiosis (Cosme et al. 2018). Another limit to Pi tracking during AM is the difficulty to effectively transform AM-forming fungi (Harrier and Millam 2001).

Traditional techniques used to quantify P in plant tissue rely on colorimetric assays, namely, molybdenum blue and malachite green assay. Both methods can be adapted to small sample volumes and allow accurate measurements in the range of nanograms. The disadvantages are the destruction of the plant tissues’ integrity and the inability to distinguish between the different pools of P. Conversely, ion chromatography allows real-time detection of orthophosphate and pyrophosphate pools but suffers from interference with ions like iron (III) and aluminum (III) (Wieczorek et al. 2022). The first-generation of FRET sensors for Pi were FLIPPi (Gu et al. 2006) (Table 2). FLIPPi consists of a Pi PBP, with the FRET pair eCFP/Venus fused into 1 lobe each. Mutations in the binding pocket generated a sensor series with affinities from *K_d_* = 260 to 30 mM. Two members of the family, FLIPPi-4µ and FLIPPi-30m, were recently expressed in Arabidopsis roots (Assunção et al. 2020). However, their short dynamic range and erratic fluorescence responses of FLIPPi-30m hinder their potential in plants (Mukherjee et al. 2015). The second-generation sensors (cpFLIPPis) switched Venus FP of FLIPPis for a cp version and introduced point mutations in the binding domain, enhancing the dynamic range (2.5-fold) and with *K_d_* of 0.08 to 11 mM (Mukherjee et al. 2015). By expressing cpFLIPPis in Arabidopsis, it was possible to estimate [Pi]_cyt_ in Pi-repleted root epidermal cells at ∼6 mM (Mukherjee et al. 2015) (Table 1). cpFLIPPi was targeted to plastids by fusing an N-terminal ribulose bisphosphate carboxylase small subunit (RbcS) chloroplast transit peptide. Expression in wt and plastid Pi transporter mutant *pht4;2* established that PHT4;2 is involved in Pi export from plastids. In a follow-up study, cpFLIPPi6.4m was expressed in leaves, in the cytosol and chloroplasts. Due to donor quenching by leaf pigments, it was suggested that the FRET/acceptor ratio is the most robust method for evaluating sensor behavior in green tissues (Banerjee et al. 2016). Quantification of Pi in roots under different nutrient conditions, coupled to absolute calibrations of the sensor by microinjection of known Pi solutions, revealed Pi ranges from 3 mM (in meristematic zone after Pi starvation) to 12 mM (in transition zone under Pi-replete conditions) (Sahu et al. 2020) (Table 1). Recently, cpFLIPPis were used to monitor relative Pi levels in the cytosol and plastids of individual cells, and responses to external Pi application, in mycorrhized root of *Brachypodium distachyon* (Zhang et al. 2022). cpFLIPPis showed that cytosolic Pi levels are higher in root cells colonized by the fungus than adjacent cells, and higher in colonized cortical cells than in epidermal cells. In contrast to plastid Pi, which did not vary following exogenous Pi pulse, cytosolic Pi in colonized cells showed a transient increase, suggesting Pi uptake via direct route. These results in both Arabidopsis and *B. distachyon* show that cpFLIPPis are suitable for real-time studies in plants.

## Potassium (K)

K is 1 major determinant of crop yield. K is involved in carbohydrate synthesis, photosynthesis, flowering, and regulation of the osmotic potential of plant cells (Hawkesford et al. 2012). The lack of K is associated with appearance of dark spots on leaves and reduces the ability to withstand dryness, frost, or fungal attack (de Bang et al. 2021). K is the most abundant cation in plant tissues, comprising up to 10% of dry matter (Mengel et al. 2001), and has high mobility across cell/tissues and long distance vascular transport (Hawkesford et al. 2012). Soil [K] is low (0.01 to 20 mM), and mostly bound to clay or present as K-minerals, with only ∼0.1% to 0.2% bioavailable (Mengel et al. 2001). Yet, plant cells present high K concentrations in the cytosol (100 to 200 mM), vacuole (10 to 500 mM), and chloroplasts (100 to 200 nM) (Hawkesford et al. 2012) (Table 1). Plants have a high-affinity uptake system acting with low K levels, and a low-affinity uptake system operating under higher K levels (Wang and Wu 2013). The lack of available K cations and low mobility in soil, coupled with extensive and recurrent droughts, are major setbacks to agriculture. A huge step has been the discovery of the K-sensing niche, explaining how K deprivation is sensed, signaled, and connected with plant development (Wang et al. 2021).

Dyes are available to measure cellular K content. One of the first used is the UV-excitable PBFI (K binding benzofuran isophthalate), which has a *K_d_* = 8 mM (Minta and Tsien 1989). More recently, a red-shifted fluorescent dye named APG-1 (Asante Potassium Green-1) was developed for screening K^+^ content in mammalian astrocytes (Rimmele and Chatton 2014). Further improvement of APG-1 led to APG-4, which is highly sensitive to K and is affected by cell volume (Rana et al. 2019). NK3 is a fluorescent indicator with high K^+^ selectivity, is stable at pH 6 to 8, and has been used to investigate K dynamics in Arabidopsis root hairs (Sun et al. 2020). Another approach to quantify K^+^ cell content involves K^+^-selective (µ)electrodes, which however are an invasive technique (Miller et al. 2001).

Genetically encoded K ion indicators (GEPIIs) were first introduced in bacteria and mammalian cells (Bischof et al. 2017) (Table 2). GEPIIs are FRET sensors composed of a chimeric protein, in which a bacterial K^+^-binding protein (KBP) is fused to the optimized cyan (mseCFP) and yellow (cpV) FPs at the N- and C-termini. To decrease K^+^ sensitivity, either acidic amino acids within the lysine motif (LysM) domain were mutated, or flexible linkers of variable length (7, 10, or 15 amino acids) were introduced between the transport-associated and nodulation (BON) and LysM domains. Recently, GEPII K sensors were successfully implemented in planta (Wang et al. 2021). Surprisingly, sensors enabled the differentiation of root tissue specificity towards K^+^ under low and sufficient [K]. This has shown that K^+^ distribution in roots is heterogenous, with the highest concentration in mature vasculature tissue, the second highest concentration in the meristematic tissue, whereas the lowly concentrated postmeristematic zone is recognized as a K^+^-sensing niche. KRaION59 (K^+^ ratiometric indicator for optical imaging based on mNeonGreen 1) uses a similar approach, with a bacterial KBP inserted into the FP. The first version of the ratiometric indicator KRaION1 exhibited a *K_d_* = 69 ± 10 mM, with sensitivity to pH fluctuations. Introduction of mutations within and around the binding site has provided *K_d_*s = 39 to 112 mM. The KRaION indicator was expressed in HeLa cells, with *K_d_* values differing from in vitro experiments, implying that further characterization under physiological conditions is necessary. Further development of K indicators based on a bacterial KBP led to KIRIN and GINKO sensors (Shen et al. 2019). KIRIN was developed in 2 forms: a cyan-yellow K^+^ ratiometric indicator (KIRIN1), and a green-red (KIRIN1-GR) FRET-based K^+^ indicator. Compared to GEPII, KIRIN1 has a lower *K_d_* = 1.66 mM, whereas the red-shifted KIRIN1-GR has a *K_d_* = 2.56 mM. GINKO1 has been engineered as a single fluorescent sensor with both intensiometric and excitation-ratiometric properties. Its design includes a bacterial KBP inserted into cpEGFP, responding to [K^+^] from 3 to 100 mM. Rational engineering led to brighter and more sensitive GINKO2 used to visualize [K^+^]_cyt_ dynamics in planta with a range from 1 to 100 mM (Wu et al. 2022b). To date, sensors that target K in the cytosol have been introduced in bacteria (Wu et al. 2022b) and mammalian cells (Wu et al. 2022b) but few in planta (Wang et al. 2021; Wu et al. 2022b). One drawback is the sensitivity to pH, which renders the use of K^+^ sensor in vacuoles difficult (pH 5.5). Also, sensitivity towards pH changes might impact real-time cytosolic K^+^ sensing, especially since pH shift may be associated with environmental changes.

## Sodium (Na)

Despite being an abundant ion in plant cells, only a minority of plants (halophytes) tolerate Na concentration above 200 mM in the soil (Maathuis 2014). Under physiological conditions, the Na concentration in tobacco plant cytosol is 1 to 10 mM (Binzel et al. 1988). Maintenance of sodium levels in cells is regulated by salt overly sensitive (SOS) loci, which includes the plasma membrane–located Na^+^:H^+^ antiporter (SOS1), kinase CIPK24 (SOS2) responsive to phosphorylation of SOS1, and CBL calcium sensor CBL4 (SOS3) (Maathuis 2014).

Traditionally, assessment of Na^+^ content in tissues is done by flame photometry method following sample acid digestion (Pauline and Hald 1946). Na selective (µ)electrodes can also be employed for measurements in plants (Carden et al. 2001) and have shown to have high selectivity and being insensitive to changes in total ionic strength and physiological ranges of pH (Carden et al. 2001). To achieve higher spatiotemporal resolutions within tissues, Na^+^ fluorescent dyes could be used. CoroNa Green AM is a cell-permeant, green-fluorescent dye, with absorbance/emission at 492 nm/520 nm (Meier et al. 2006). It has been employed to study Na^+^ uptake in Arabidopsis (Park et al. 2009) and wheat (Wu et al. 2018) roots. The Na^+^-sensing fluorescent probe sodium-binding benzofuran isophthalate (SBFI) is another dye that allows visualization of Na uptake (Meier et al. 2006). Cell-permeable SBFI-AM has *K_d_* = 3.8 mM in the absence of K^+^ and 11.3 mM for solutions with combined Na^+^ and K^+^ concentration of 135 mM (physiological ionic strength). It has ∼18- times more selectivity for Na^+^ than for K^+^. SBFI-AM has been applied to study Arabidopsis root hairs under salinity stress, showing that cells are able to accumulate 15 to 60 mM of Na^+^ depending on concentrations in the medium (Halperin and Lynch 2003). Currently, no Na-specific sensor is available.

## Magnesium (Mg)

Mg is crucial for plant growth and development; it is necessary for photosynthesis, phloem loading, leaf senescence, and stomata opening and acts as an enzyme activator (Tian et al. 2021). Mg contributes also to sugar storage. Mg deficiencies lead to weak stalks, yellow and brown spots on leaves, and loss of greenness (de Bang et al. 2021). Mg is abundant in plant cells with varying concentrations across the cellular compartments, from 0.2 to 0.5 mM in the cytosol and mitochondria, 10 to 15 mM in chloroplasts, and up to 5 to 80 mM in vacuoles (Hermans et al. 2013) (Table 1). Mg accumulates in tissues in several biochemical forms. Although chlorophyll synthesis in photosynthetic tissues requires a substantial pool of Mg^2+^ (20% of total Mg^2+^ is in chloroplasts), it can increase to 50% under low light conditions, leading to Mg^2+^ remobilization (Tian et al. 2021). [Mg] has been determined using techniques like Inductively Coupled Plasma Optical Emission Spectrometry (ICP-OES) and indirect measurements of relative chlorophyll concentration (Bischof et al. 2017). Measurements of plant intracellular Mg^2+^ have been reported using Mg-binding ionophores and fluorescence spectrophotometry. However, many Mg^2+^ fluorescent probes lack specificity. 31P-NMR cells were used (Table 1). Other methods like X-ray microanalysis (XRMA) and 13C-NMR citrate/isocitrate ratio have been used (Tian et al. 2021) as indirect measurements which report only average quantities. Synthetic dyes have allowed Mg determination but have limited specificity due to possible Ca^2+^ binding at micromolar range (Kim et al. 2007). One exception is KMG-104, an intensiometric dye reported to be highly specific to Mg^2+^ (Komatsu et al. 2004). Other ratiometric Mg^2+^ dyes include Mag-Fura and Mag-Indo, which however, required cytotoxic UV excitation (Tränkner and Jaghdani 2019).

Various Mg^2+^ sensors have been reported. MagFRET is based on the high-affinity binding domain of human centrin 3 (HsCen3) fused to cerulean and citrine FPs. Affinity variants of MagFRET have been used to monitor [Mg^2+^]_cyt_ in HEK293 mammalian cells (Lindenburg et al. 2013). A newer sensor, MagIC (Koldenkova et al. 2015), is composed by a YFP fused to a reference red–emitting FP allowing ratiometric imaging (Lindenburg et al. 2013) (Table 2). MagIC was distributed between the cytosol and nucleus, but its fusion with a signal peptide targeted it to the mitochondria or endoplasmic reticulum (ER). This sensor is promising for monitoring cellular Mg^2+^ in planta, taking advantage of its millimolar-order affinity.

## Calcium (Ca)

Ca has several physiological roles, such as countercation for organic and inorganic anions, activates plant growth–regulating enzymes, helps in nitrate assimilation, and plays roles in crosslinking pectin in cell walls and in cell plate formation during cell division (de Bang et al. 2021). As a second messenger, Ca contributes to signaling during abiotic and biotic stress (Lee and Seo 2021). The contribution of Ca to plant physiology extends further to root–microbe interactions, where Ca^2+^ signaling is needed in the formation of root nodules in legumes and their colonization by (N)-fixing bacteria (Lévy et al. 2004). Most Ca^2+^ is in the apoplasm, in the range of 0.3 to 1 mM, and even up to 10 mM (Luan and Wang 2021). Within cells, the vacuole and ER are the largest Ca pools (200 *µ*M to 50 mM and 50 to 500 *µ*M, respectively) (Table 1). [Ca^2+^]_cyt_ is actively maintained at ∼100 nM, with free Ca^2+^ buffered by Ca-BPs and active export to the apoplasm, vacuole, and ER (White and Broadley 2003; Plattner and Verkhratsky 2015). Divergent mechanisms exist that mediate Ca^2+^ influx to the cytosol, resulting in transient, often oscillatory elevations of [Ca^2+^]_cyt_. Studies have extensively dealt with roles of Ca^2+^ in signaling (Costa and Kudla 2015, p. 2). Here, we provide an overview from a nutrient perspective. Although Ca deficiencies are not as common as for other nutrients (White and Broadley 2003), a shift in Ca available in soil can influence plant performance.

Intracellular [Ca^2+^] can be quantified. Numerous Ca^2+^-sensitive dyes have been generated and successfully utilized in planta (Box 2). Many genetically encoded Ca sensors have also been developed over the past 30 yrs, with the most important included in Table 2. The first Ca^2+^ sensors were based on Aequorin (Knight et al. 1991), a holoenzyme that requires the external application of its prosthetic group coelenterazine. Improvement was made with FRET–based Cameleon sensors, which allow higher sensitivity (60% to 80%) and spatiotemporal resolution. Cameleons were designed as CFP and YFP linked by calmodulin (Ca^2+^ BP, CaM) and the M13 peptide (Miyawaki et al. 1997). Their ratiometric design has been utilized to quantify Ca^2+^ dynamics in cellular compartments (Allen et al. 1999b; Costa et al. 2018). Further development of Ca sensors led to higher signal-to-noise ratios and sensitivity in planta, with single-FP sensors like GECOs (Waadt et al. 2017) and GCaMPs (Akerboom et al. 2009). More improvements were made later by introducing a reference FP (Ast et al. 2017). The latest advances have led to a new series of Ca sensors named NEMO, derived from mNeonGreen, which show fast kinetics and ultrawide dynamic ranges (in cellulo over 100-fold), with *K_d_* of 141 to 557 nM in vitro (Li et al. 2022). In vivo measurements with NEMO sensors outperformed most of the existing state-of-the-art GECIs. Ratiometric Ca indicators could be used to quantify intracellular [Ca^2+^]. However, it usually requires in vivo calibration with Ca ionophores or chelators (Sadoine et al. 2021b). Studies have used Ca sensors targeted to Ca^2+^ channels in order to measure Ca transport activity and, thereby, uncovered confined cytosolic zones in their vicinity termed Ca^2+^ nanodomains (Tay et al. 2012). These examples show the potential of sensors to improve our understanding of cellular dynamics and organization.

Box 2. **40 yrs of engineering of Ca^2+^-sensitive dyes.**

Numerous Ca^2+^-sensitive dyes have been generated over the past 40 yrs and were successfully utilized to quantify Ca concentrations in planta. Fluorescent dyes for Ca^2+^ can be classified into ratiometric and nonratiometric (Johnson et al. 2011). The dyes interact with intracellular Ca^2+^ through a cage of carboxylic acid groups (Swanson et al. 2011), which leads to change in fluorescence intensity and/or excitation maximum. Among nonratiometric dyes, Calcium Green-1 has been an extremely useful qualitative indicator for resolving spatial Ca^2+^ distribution in roots and stomata (Wang et al. 2016). However, it does not allow quantitative measurements of Ca^2+^, it is prone to photobleaching, and its distribution within different cell types may be uneven (Swanson et al. 2011). Other available nonratiometric dyes include Fluo-2, -3, and -4; Calcium Green-2; and Rhodamine-based indicators (Kanchiswamy et al. 2014). Ratiometric dyes offer quantitative measurements of cellular Ca dynamics. The advantage of these molecular probes, aside from their use in quantitative imaging, is the possibility to correct for uneven dye loading, photobleaching, and focal plane shifts, as well as the availability of different *K_d_* ranges and spectral properties, making them easily adjusted to a particular microscopy setup (Kanchiswamy et al. 2014). Among the many probes, 2 commonly used bright indicators with similar *K_d_* values (∼250 nM) are Fura 2, with excitation peaks at 340 and 380 nm that allow excitation ratiometric imaging, and Indo-1, with excitation peaks at 405 and 485 nm that are suitable for emission ratiometric imaging (Wu et al. 2022a). To date, Fura 2 and Indo-1 have been used to quantify the cytosolic Ca transients in Arabidopsis root hairs, guard cells, pollen tubes, and protoplasts. The sensitivity of a probe to acidic pH might pose a limiting factor in cases where it is necessary to target the probe to compartments such as the vacuoles, vesicles, or the cell wall area (Sadoine et al. 2021a). In addition to pH, tissue geometry and autofluorescence vary between compartments, making it necessary to look for FPs with matching properties that allow unhindered imaging (Donaldson 2020). Since not all plant species can be successfully transformed to express fluorescent sensors, Ca^2+^-sensitive dyes are highly important to exploring cellular Ca dynamics (Swanson et al. 2011). Yet, several disadvantages for introducing fluorescent dyes into eukaryotic cells include the need for permeabilization of the cell wall and plasma membrane (Johnson et al. 2011), the nonreversible binding of the dye to Ca^2+^, and dye accumulation within organelles (Swanson et al. 2011).

## Sulfur (S)

As an essential element required by all living organisms, S is 1 of the most versatile nutrients in plants. It is involved in reductive iron assimilation, photosynthesis, and oxidation–reduction homeostasis. S is present in the amino acids methionine and cysteine and in metabolites such as glutathione (GSH), vitamins (e.g. biotin and thiamine), chlorophyll, and coenzyme A. Additionally, S plays important roles in responses to biotic and abiotic stresses. Notably, GSH eliminates reactive oxygen species under oxidative stress (Li et al. 2020) and glucosinolates are S-containing secondary metabolites involved in defense mechanisms (Zhang et al. 2020). Reactive S species (RSS) are molecules that play important roles in physiological processes via protein sulfhydration (Liu et al. 2021). Hydrogen sulfide (H_2_S) is a gas that recently has been found to be involved in various physiological activities and different signaling pathways (Liu et al. 2021). In plants, H_2_S is involved in seed germination, growth, development, and tolerance to abiotic stress such as salinity, drought, and extreme temperatures. There are 2 possible routes for S absorption: via root uptake through S transporters in root epidermal cells, or via stomata by gas exchange. Subsequently, S is loaded into the xylem and distributed throughout the plant, where it can be stored in vacuoles or transported to chloroplasts (Buchner et al. 2004). Most of S assimilation into amino acids occurs in chloroplasts of young leaves, although assimilation and production of cysteine can occur in seeds and roots (Takahashi et al. 2011).

Measuring intracellular concentrations of S species in vivo is challenging especially when they are present at low concentrations and have high reactivity and short lifetimes and no direct chemical tools are available. For RSS, fluorescence bioimaging in real-time detection of S species allows intracellular real-time detection (Yu et al. 2015). Recently, a novel sulfane S sensor NIR fluorescent probe, SSNIP, has been developed that displays enough accuracy and reliability to monitor sulfane S in Arabidopsis roots at different developmental stages (Jiang et al. 2019). A ratiometric sensor for H_2_S (hsFRET) had been generated and tested to monitor H_2_S in living cells (Youssef et al. 2019). However, hsFRET requires exogenous addition of p-azidophenylalanine (pAzF) therefore limiting its use.

## Chloride (Cl)

Cl is mostly present as a highly mobile free anion with shoot concentrations ranging from 28 *µ*M to1.68 mM (Broadley et al. 2012). At high concentrations, Cl can be toxic, leading to chlorosis and necrotic lesions, symptoms of leaf-tip burning (Geilfus 2018). Increasing soil salinity is a growing problem worldwide due to irrigation practices and climate change (Van Zelm et al. 2020), threatening crop production. In barley, vacuolar [Cl^−^] is 50 to 150 mM, while phloem concentration can be up to 120 mM (Xu et al. 1999; Broadley et al. 2012).Typically, [Cl^−^] is assayed using chloride analyzer devices, which can determine the total Cl^−^ content in samples. Other approaches involve inductively coupled plasma mass spectrometry (ICP-MS) (Wheal and Palmer 2010) and Cl^−^ selective (µ)electrodes (Li et al. 2017a). Fluorescent dyes like 6-methoxy-N-(3-sulfopropyl)quinolinium (SPQ), N-(ethoxycarbonylmethyl)-6-methoxyquinolinium bromide (MQAE), and 6-methoxy-N-ethylquinolinium iodide (MEQ) are available (Arosio and Ratto 2014). These dyes are nonratiometric are quite insensitive to bicarbonate concentration and pH variations and have fast kinetics (Arosio and Ratto 2014). Several Cl sensors exist (Table 2), based on the intrinsic sensitivity of FP proteins to halide presence. The main limitation of current Cl sensors is their sensitivity to pH (Waadt et al. 2020) and low affinity for Cl (Lodovichi et al. 2021). YFP is the simplest version, since halide can directly bind YFP variants and modulate their fluorescence emission (Bregestovski and Arosio 2011). A ratiometric FRET version was created in Clomeleon, attaching a Cl^−^-insensitive CFP to a Cl^−^-sensitive YFP variant. Despite that in neurons, it proved not sensitive enough (Bregestovski et al. 2009; Bregestovski and Arosio 2011), its high *K_d_* = 160 mM would fit well Cl concentrations in plant cells. Accordingly, Clomeleon has been used to screen Cl^−^ dynamics in Arabidopsis roots exposed to NaCl stress (Lorenzen et al. 2004), unraveling the root Cl^−^ influx under high salt concentrations. The studies showed that Cl^−^ influx under saline conditions occurs passive through channels and is dependent on the presence of external Ca^2+^ (Bregestovski et al. 2009; Bregestovski and Arosio 2011). The Cl^−^ sensor, similar to Clomeleon, is based on the coupling of CFP and YFP with a peptide linker and exhibits a *K_d_* = 30 mM, although it is still sensitive to pH changes (Bregestovski and Arosio 2011). ClopHensor is a ratiometric sensor based on a GFP variant which permits simultaneous real-time detection of pH and Cl^−^. It is designed with monomeric DsRed as reference fluorophore fused to E2GFP, with a *K_d_* = 50 mM at physiological pH (7.2 to 7.3) (Bregestovski and Arosio 2011). ClopHensor is a triple excitation and dual-emission sensor, in which the 458 nm excitation of E2GFP is pH independent and chlorine sensitive, the 488 nm excitation of E2GFP is pH dependent, and the 543 nm excitation of DsRed monomer is independent of pH and Cl^−^ (Bregestovski and Arosio 2011). ClopHensor has been expressed in the cytoplasm of Arabidopsis guard cells (Demes et al. 2020). The reported *K_d_* = 17.5 to 163 mM values of ClopHensor are higher than the physiological cytosolic Cl^−^ levels (∼10 mM) (Felle 1994), and it appears to function also as a nitrate sensor (Demes et al. 2020). Recently, the ChlorON sensor series has been developed, in which mNeonGreen was transformed into turn-on fluorescent sensors for Cl (Tutol et al. 2022) with large dynamic ranges and varying affinities for chloride (*K_d_*s = 30 to 285) and other ions like bromide, nitrate, and iodide. Among Cl sensors that have been used in planta, E2GFP was successfully expressed together with a Ca sensor (R-GECO1-GSL-E2GFP) to simultaneously monitor Ca^2+^, H^+^, and Cl^−^ dynamics, highlighting a remarkably high spatiotemporal overlap in response to IAA, ATP, and glutamate (Waadt et al. 2020). Translation fusions are prone to FRET artifacts, with sensors being in close proximity to each other. Preventing the FRET to occur may be done by using a 14–amino acid ASGGSGGTSGGGGS-linker (GSL), or the self-cleaving 22–amino acid P2A linker (Waadt et al. 2020; Li et al. 2021).

## Iron (Fe)

Fe is essential for chlorophyll production in plants and serves as cofactor for many enzymes and redox systems. It is involved in energy transfer, N reduction and fixation, and lignin formation (Marschner 1998). Fe deficiency causes yellowing between the veins of younger leaves (Lucena and Hernandez-Apaolaza 2017). Fe can be toxic when it accumulates within cells; therefore, Fe homeostasis is carefully regulated. Plants have developed different strategies for Fe uptake and transport. Organelles such as chloroplasts and mitochondria play a central role in Fe economy since Fe is a key cofactor for many enzymes involved in the electron transport chain and photosynthetic complexes (Vigani et al. 2013). Fe is highly reactive and must be chelated to avoid cellular damage. Monocotyledons, such as barley, rice, and maize, adsorb Fe(III) and dicotyledons, such as Arabidopsis and tomato, and absorb Fe(II) from the soil (Connorton et al. 2017). Plants encounter major issues with Fe uptake/transport as free ion, its toxicity, and insolubility. The latter can be overcome by inducible chelation and reduction systems at the root surface that facilitate the uptake of iron, e.g. in tomato and Arabidopsis, Fe^3+^ is reduced by Ferric reduction oxidase 2 (FRO2) to Fe^2+^ before being transported into cells through Fe-regulated transporter 1 (IRT1) (Connorton et al. 2017). In cells, Fe is bound to specialized proteins before being integrated into molecules (Hell and Stephan 2003).

In situ experiments have been performed using an Fe pyoverdine-doped sol–gel glass, taking advantage of a combination of fluorescent compounds that undergo quenching in response to a specific analyte and a Fe-specific siderophore, pyoverdine from *Pseudomonas spp* (Yoder and Kisaalita 2011). However, no real-time monitoring of Fe in organelles has been performed yet. To date, no Fe sensor has been developed.

## Boron (B)

B is involved in membrane stability, sugar transport, cell wall formation, amino acid production, and flowering (Shorrocks 1997). B is toxic when present in excess, a worldwide problem for food production, especially in arid areas. Soils with insufficient B are also common leading to wilted growing points, stunted growth, leaf deformation, and poor flowering (Koshiba et al. 2009a). B is an uncharged small molecule that can transit by passive diffusion across membranes. However, when availability is limited, plants use transporters to acquire sufficient levels of B (Koshiba et al. 2009b). Phloem mobility depends on species, and in most cases, B binds to sugar alcohols for reallocation from old to young leaves (Hu et al. 1997).

B measurements in Arabidopsis roots have been reported by ICP-MS and laser ablation–ICP-MS (Shimotohno et al. 2015). Cytosolic [B] in different plant tissues following B treatment has been monitored to resolve [B] spatial distribution and temporal dynamics using sensors based on the uNIP5;1-Venus/Luc system (Fukuda et al. 2018).

## Manganese (Mn)

Mn is involved in respiration, N assimilation, germination, fruit ripening, and pathogen resistance (Tao et al. 2023). Mn is also essential for photosynthesis as a part of the metalloenzyme cluster of the oxygen-evolving complex (OEC) in photosystem II (PSII). It is absorbed by plants as Mn^2+^, and cell levels are ∼20 to 40 ppm (mg kg^−1^) dry weight (Alejandro et al. 2020). Mn homeostasis is therefore highly regulated during uptake, distribution, allocation, and storage (Alejandro et al. 2020). Mn transporters have only recently been characterized in a wider range of plant species (Alejandro et al. 2020). Mn^2+^ is acquired from soil by roots through transporters including NRAMP, ZRT/IRT, and YSL (Alejandro et al. 2020). The main pathway for Mn translocation and distribution is towards the xylem, to the phloem where mobility is low, and to tissues (Li et al. 2017b). It is also suggested that Mn^2+^ could be taken up via leaf cells in rice (Sasaki et al. 2012).

Mn has been measured in plants (Leplat et al. 2016) by atomic emission spectroscopy or ICP-OES (Leplat et al. 2016). Sensors for Mn have been reported including the fluorescent Mn sensor M1 and iLovU (Table 2), that is based on photo-induced electron transfer (Liu et al. 2014). To date, however, no Mn sensor has been employed in plants.

## Zinc (Zn)

Zn is required for growth, and it is involved in biological processes like cell proliferation, carbohydrate metabolism, and P-Zn interactions (Rehman et al. 2012). The Zn deficiency phenotype depends on the plant type, but common symptoms include stunted growth, reduced internode length, smaller young leaves, and yellowing of lower leaves. Zn is present in ∼8% to 10% of all eukaryotic proteins (Andreini et al. 2006). It serves as a cofactor in the catalytic domains of enzymes like carbonic anhydrase, alcohol dehydrogenase, and alkaline phosphatase and as a structural scaffold for proteins including Zn finger transcription factors (Clemens 2022). It may also serve as a facilitator of protein–protein interactions (Kocyła et al. 2021). Therefore, along with Fe, Zn is the most ubiquitous microelement in all proteomes. Unlike Fe, Zn has only a single oxidation state and does not form free radical species, making it ideal for proteins that interact with nucleic acids, such as transcription factors. Estimates of [Zn] within a cell are of 100 to 500 *µ*M. Most of Zn is bound to proteins, with only 50 to 500 pM available as a free pool (Clemens 2022). However, it is likely that “free” Zn atoms are also associated with low-molecular weight–buffering ligands (Clemens 2022). It is believed that free Zn concentrations are even lower in organelles, ranging at subpicomolar levels. Zn can either be covalently bound or be loosely associated in proteins. A proteome analysis of yeast revealed that 37% to 57% of Zn-associated proteins reside in the nucleus (Wang et al. 2018). However, approximately 90% of Zn atoms are localized in the cytosol, since many ribosomal proteins carry Zn. Conversely, only 3% of Zn is present in the nucleus, due to the low abundance of transcription factors (Wang et al. 2018; Clemens 2022).

Traditional techniques for metal quantification, like atomic spectroscopy, are destructive, offer no subcellular resolution, and do not discriminate between free and protein-bound Zn. Chemical probes offer better alternatives, reviewed in Clemens 2022. Their design consists of an organic fluorophore coupled to a Zn chelating agent. In the absence of Zn, the metal-binding moiety quenches the fluorophore. When Zn is bound, quenching is relieved and fluorescence emission increases. Chemical probes usually offer a large dynamic range, but poor subcellular resolution. Some probes spontaneously localize in specific organelles, such as the Golgi for FluoZin-3 and ZP1, and the ER for ZBR1–3. Decoration with functional groups has been used to target probes to lysosomes, mitochondria, and cell surface, with mixed results. Additionally, the accumulation of probes within the cell can disrupt Zn homeostasis, chelating the small free Zn pool, thus unbalancing the cell physiology (Clemens 2022) similar to a “sponge effect” (Castro-Rodríguez et al. 2022).

Zn sensors have been developed, employing both FRET-based and intensiometric approaches (reviewed in Pratt et al. 2021; Table 2). The FRET-based Zn sensors belong to 3 families: ZapCY, eCALWY, and eZinCh. They differ in their Zn binding domain: ZapCYs employ the yeast Zn finger transcription factor Zap1 (Qin et al. 2011); eCALWYs use the metal binding domains of Atox1 ATP7B (WD4) linked by a long flexible linker (van Dongen et al. 2007; Vinkenborg et al. 2009); and eZinChs were developed by modification of the FP pair. The latter differs conceptually as it introduces Zn binding residues directly on the FP surface to convert it into a Zn sensor (Evers et al. 2007). However, eZinChs and CALWYs also react to cadmium, lead, and cobalt, which are transition metals with properties similar to Zn (van Dongen et al. 2006). Conversely, ZapCY only reacts to Zn (Qin et al. 2011). eCALWY and eZinCh members have also been converted into BRET (bioluminescence resonance energy transfer) sensors, through addition of a luciferase domain (Aper et al. 2016). Compared to FRET, BRET has reduced phototoxicity, light scattering, and autofluorescence, frequent issues in plant tissues. ZapCY, eCALWY, and eZinCh are present in variants with affinities spanning from 2 pM for ZapCY1 and eCALWY1 to 811 pM for ZapCY2, 2.3 nM for eZinCh8, and 2.9 nM for eCALWY6 (Qin et al. 2011). Those sensors are able to detect Zn fluctuations in mammalian cell cytosol. The 3 sensor families have also been expressed in the ER with early reports on Zn concentrations that were contradictory, as ZapCY1 displayed lower ER [Zn] compared to the cytosol, while eCALWY4 showed higher values (Qin et al. 2011). Additionally, ZapCY1 was the sensor showing the least consistent signal in the ER, in terms of variation in dynamic range and FRET ratio. Conversely, ∼25% of cells transformed with eZinCh2 showed dotted patterns indicative of distortion of the ER structure. Another recently developed FRET sensor is Zn72R, which is based on the Zn binding RING motif from the protein TRIM72 coupled to a FP pair. However, its low affinity (56 *µ*M) and low dynamic range make it only suitable for cell compartments rich in Zn, such as insulin granules, and are probably less useful in plants (Xian et al. 2020). Intensiometric sensors for Zn have been developed. GZnPs are cognate single-FP versions of ZapCY, since they are also based on Zn finger motifs from Zap1 (Fudge et al. 2018). The addition of a reference FP, mCherry, to the N-terminus makes GZnP2 ratiometric. GZnP1 and GZnP2 have affinities in picomolar range (58 and 352 pM, respectively) and large dynamic ranges (2.6 and 4.5, respectively), making them suitable for plants. Other single FP sensors are ZnGreens, ZnRed, and ZIBGs, which differ in the FP used (TFP, Apple, and GFP, respectively; Chen and Ai 2016). Although their dynamic ranges are high (up to 26.3 for ZnGreen1), their affinities for Zn are too low (micromolar and nanomolar ranges) to be used in plants. Lanquar et al. (2014) stably expressed 5 eCALWY variants (1 to 4 and 6, with *K_d_* = 1.8 pM to 2.9 nM) in the silencing-resistant Arabidopsis mutant *rdr6* (Lanquar et al. 2014). Steady-state measurements revealed that in roots, the cytosolic [Zn] is 420 ± 200 pM for plants grown in normal conditions (5 *µ*M Zn supply), increasing to 2,000 ± 600 pM when plants are grown in media with excess Zn (30 *µ*M). This indicates buffering capacities of plant cells in the picomolar to nanomolar ranges, even when external concentrations are 3 to 4 orders of magnitude higher (Lanquar et al. 2014). Interestingly, dynamic experiments with a microfluidic device have shown that cells depleted of their free Zn pool (by addition of a chelator) are able to slowly refill their pool using either high-affinity transporter or Zn release from intracellular stores (Lanquar et al. 2014).

## Copper (Cu)

Cu is a redox-active transition element required for enzymes like ascorbate oxidase, superoxide dismutase, and cytochrome-C oxidase. Cu is also important in chloroplasts for quinone synthesis (Broadley et al. 2012). Approximately 260 Cu-dependent proteins are expressed in Arabidopsis (Schulten et al. 2019). Also, Cu is a cofactor in the ER–localized ethylene receptor ETR1, conveying specificity to binding the small ligand (Rodrıguez et al. 1999)

Cu deficiency can lead to yield loss in small grains (Yruela 2009). Plants acquire Cu from soil and then transport it to different tissues. The critical free [Cu] in nutrient media (below which deficiency occurs) ranges from 10^−14^ to 10^−16^ mM. Soil concentrations of Cu typically range from 10^−3^ to 10^−6^ mM (Yruela 2009), and the Cu content in plant tissue is approximately 10 *µ*g g^−1^ of dry weight.

Traditional techniques for detecting Cu in plants include destructive methods such as atomic absorption spectrometry (AAS) or ICP-OES. In addition, several fluorescent dyes can be used for quantifying Cu. Phen Green FL is able to detect both Cu^2+^ and Cu^+^, as well as other ions (Rakshit et al. 2018). Phen Green SK is more selective and can be used in conditions where oxidation of Cu^+^ to Cu^2+^ is minimized (Shingles et al. 2004). The BODIPY-based Copper sensor 1 (CS1) has 10-fold turn-on response and high selectivity for Cu (Cotruvo et al. 2015). CS1 has been used to detect Cu in Arabidopsis and helped to identify the genes FRO4/FRO5 involved in Cu root accumulation (Bernal et al. 2012). An improved version of CS1, Copper sensor-3 (CS3), exhibits improved 75-fold turn-on response with a higher quantum yield for the Cu(I)-bound sensor, in order to maintain visible excitation/emission profiles (Cotruvo et al. 2015). CS3 was used to screen Cu sequestration in cells of Zn-deficient *Chlamydomonas reinhardtii* green algae (Hong-Hermesdorf et al. 2014). Other dyes are available, such as Copper sensor-790, Rhodol-based Cu fluorophores, and FluTPA1 (Cotruvo et al. 2015). CusSR is a Cu reporter probe, and it acts as part of the 2-component system for detection of Zn and Cu (Ravikumar et al. 2012). The CusSR probe was designed by fusing the Cu-responsive promoter (cusC) to the RFP gene, and it senses Cu with a detection limit of 26 *µ*M. However, this sensor is associated with an unstable Cu ion trapping process, caused by disruption of membrane integrity by overexpression of ompC proteins and toxic metal accumulation in cells. So far, there is only 1 published study on Cu probe in planta, which aimed to establish a phytoindicator tool for Cu-contaminated soils. This technique is based on the Cu-inducible gene expression system, in which the first element, a chimera of the yeast transcription factor activating a Cu–metallothionein expression (ACE1), is fused with the VP16 activation domain (VP16AD) of the herpes simplex virus; the second element is GFP (Saijo and Nagasawa 2015). The reporter system was utilized for screening soil-bioavailable Cu, and GFP fluorescence increased with increasing [Cu] from 20 to 500 mg kg^−1^ (Saijo and Nagasawa 2015).

Sensors for Cu have been tested mostly in bacteria, yeast, and mammalian cells (Table 2). The Amt1-FRET sensor was constructed inserting the Cu binding domain of Amt1 (residues 36 to 110) between CFP and YFP (Wegner et al. 2010). The Amt1-FRET response is specific for Cu and *K_d_* is 2.5 × 10^−18^ M (Wegner et al. 2010). Introduction of Amt1-FRET in mammalian cells showed potential for imaging Cu^+^ fluctuations in vivo. Ace1-FRET and Mac1-FRET (Wegner et al. 2011) are based on 2 opposing yeast Cu regulators, Cu(I)-binding domains of Ace1(36 to 100) and Mac1(203 to 295) inserted between CFP and YFP. *K_d_* of Ace1-FRET and Mac1-FRET are 4.7 × 10^−18^ and 9.7 × 10^−20^ M, respectively (Wegner et al. 2011). So far, Cu sensors have not been used in plants.

## Molybdenum (Mo)

Mo is a transition element (oxidation state from +2 to +6) essential for living organisms (Broadley et al. 2012). Mo is important as a catalytic center in molybdoenzymes, for oxidizing and reducing carbon, N, and S metabolites (Mendel and Kruse 2012). Mo is required for synthesis and activity of the key enzyme involved in N fixation, a vital element for symbiotic N fixation by rhizobacteria (Broadley et al. 2012). To prevent toxicity, Mo is kept inside cells at a low concentration (Broadley et al. 2012). Plants take up Mo as molybdate (MoO_4_^2−^), a weak Lewis acid whose availability depends on soil pH (Tomatsu et al. 2007). Mo is highly mobile in xylem and phloem and is likely shuttled across plant tissues as MoO_4_^2−^ (Broadley et al. 2012). In Arabidopsis, the vacuole is the main storage compartment for Mo (Gasber et al. 2011), where the MOT2 transporter exports Mo to the cytosol. Mo import is regulated by MOT1, a high-affinity molybdate transporter (Km ∼20 nM) localized at the plasma membrane of root epidermis and phloem cells (Tomatsu et al. 2007).

Total Mo concentration in plant tissues can be analyzed by the ICP-OES method, following acid digestion (Gasber et al. 2011). This method cannot distinguish between free and protein-bound elements and lacks cellular and subcellular resolution. One of the first Mo sensors, MolyProbe, was composed of the FRET FP pair CFP and YFP and the bacterial-responsive protein ModE (Oliphant et al. 2022). MolyProbe responds to Mo in the nanomolar range (*K_d_* = 4.7 × 10^−8^ M) (Anderson et al. 1997; Nakanishi et al. 2013), appropriate for intracellular measurements. However, MolyProbe also reacts to sulfate (*K_d_* = 2.7 × 10^−2^M) (Nakanishi et al. 2013). Latest efforts have led to in vitro quantifications of Mo in cell extracts of Arabidopsis and the fungi *Neurospora crassa* and *Saccharomyces cerevisiae* (Oliphant et al. 2022).

## Concluding remarks

The use of sensors in plants is key for monitoring nutrients with high spatiotemporal resolution in living cells while being minimally invasive. Despite being present for more than 3 decades with a large number of diverse sensors already available, their use in plants is yet surprisingly limited. Achievements outside plant biology have proven their utility and invite to further applications in planta, complementing traditional chemistry–based methods and bioassays, and recent studies have demonstrated the potential of combinatorial sensor application for multiplexed experimental approaches. This will not only help to shed light on kinetics of individual nutrients but also advance our knowledge of how nutrients, metabolites, and signaling interact on a physiological level and provide understanding of how plants cope with changing nutrient availability (see “Outstanding questions” and Box 3).

Box 3. **Current challenges and future perspectives.**

Measuring the content and dynamics of nutrients within the plant is challenging since quantifying spatiotemporal information in living cells remains difficult. Furthermore, most techniques destroy the cellular compartments, dilute compounds, and, if not destructive, interfere with the plant physiology. This limits plant scientists to address exciting questions that require monitoring nutrients in cells from living organisms while being lowly invasive. The use of sensors makes real-time monitoring of nutrients with high spatiotemporal resolution possible, providing information on nutrient distribution and dynamics. Notably, the ratiometric FRET-based K^+^ GEPII sensor has been used to study spatiotemporal dynamics of K^+^ in Arabidopsis roots revealing a group of postmeristematic cells constituting a K^+^-sensing niche. This demonstrates how root development and growth are fine-tuned depending on [K^+^]. Yet, this is 1 of few examples of advances reported. Information on in planta dynamics is still lacking for most nutrients, and metabolites are still lacking. Moreover, information on changing nutrient levels in different cellular compartments remains very scarce despite its importance for intracellular processes and tissue function. The modest advances in the plant field might be due to the specific challenges associated with establishing sensors in plants. In particular, autofluorescence from plant compounds can be a significant obstacle to imaging and even be exacerbated by additional accumulation of autofluorescence defense molecules. The quality of the data depends on both the sensor properties and the background fluorescence. Thus, the excitation and emission filters and the imaging system must be chosen taking it into account. For instance, a compromise between sensitivity and spatial and/or temporal resolution must be reached. While technologies are rapidly evolving, there are still limitations, e.g. due to the restrained possibilities of laser lines available. The use of affinity variants and a nonbinding control sensor is necessary for different compartments and specific cell type/organelles. Moreover, caution is needed also when applying in vitro-estimated *K_d_* to measurements performed in living cells, since significant discrepancy in affinity may exist, and the range of potential ligands that can tested in vitro is limited. Finally, the high pH dependencies of the fluorophores are a common issue which needs proper sensor control and/or simultaneous measurements of pH changes. New AI-aided approaches are promising for helping accelerate sensor design/optimization and may lead to more systematic use of sensors in important research topics such as plant–microbe interactions and symbiosis formation, advancing knowledge for improved productivity of in crops.
